# Dialysis Disequilibrium Syndrome as a Cause of Unexplained Pain in a Young Female: A Case Report

**DOI:** 10.7759/cureus.91562

**Published:** 2025-09-03

**Authors:** Terry O Derias, Peter P Wahba, Maria Khan, Catherine Boulanger, Alexis Powell

**Affiliations:** 1 Medicine, University of Miami Miller School of Medicine, Miami, USA; 2 Medicine, Florida International University, Herbert Wertheim College of Medicine, Miami, USA; 3 Internal Medicine-Pediatrics, University of Miami Miller School of Medicine, Miami, USA; 4 Internal Medicine, University of Miami Miller School of Medicine, Miami, USA

**Keywords:** case report, dialysis disequilibrium syndrome, female pain, unexplained pain, dialysis

## Abstract

Unexplained pain can be challenging to diagnose, especially when the workup is unremarkable. For this young woman, her symptoms appeared consistent with dialysis disequilibrium syndrome (DDS), a neurological sequela that occurs in the first few months after beginning dialysis. Due to the nonspecific presentation and difficulty in confirming this syndrome, physicians should be aware of this as a potential cause of pain in dialysis patients.

This case involves a 27-year-old woman with a past medical history of type 1 diabetes mellitus (T1DM), gastroparesis, hypertension, recurrent pyelonephritis, hyperlipidemia, depression, and end-stage renal disease secondary to poorly controlled T1DM (on dialysis), who presented with a three-day history of severe left flank and right upper quadrant pain, abdominal distension, nausea, vomiting, bilateral leg swelling, and headache. She stated she had been getting similar pain since she began receiving dialysis four months prior to her presentation. After emergent hemodialysis in the hospital, she developed worsening 10/10 generalized abdominal pain with severe tenderness, headache, nausea, chest pain, and palpitations. All workups came back negative. Given these findings, symptoms appeared consistent with DDS.

This patient struggled with a nonspecific array of symptoms that originally did not point toward any one diagnosis. Her symptoms would become even more severe when she missed dialysis sessions and subsequently completed her sessions a few days late, fitting the clinical picture of DDS. Once DDS is suspected, several measures can be taken to prevent symptoms, including administering hypertonic saline, utilizing hemofiltration in place of hemodialysis, and prolonging dialysis sessions.

## Introduction

This case of a young woman with severe, unexplained pain is unique in that she had been admitted to the hospital many times without a cause for her pain being found. However, throughout her hospital stay, a clear link between her pain and the timing of her dialysis sessions became evident. Her symptoms appeared consistent with dialysis disequilibrium syndrome (DDS), a rare neurological sequelae that occurs in the first few months after some patients receive dialysis. This syndrome is believed to result from the rapid correction of a uremic or hyperosmolar state, causing a wide range of symptoms, including headache, nausea, vomiting, muscle cramps, and dizziness [1]. More serious complications include seizures and death [1]. Some risk factors for developing this condition include the first hemodialysis session, sudden changes in the dialysis regimen, a blood urea nitrogen (BUN) level greater than 175 mg/dL, being a child or elderly individual, and having a pre-existing neurological condition [2,3]. This patient had changes to her dialysis regimen due to multiple hospital admissions, which may have contributed to recurring symptoms. DDS is largely underreported, and the incidence has not been clearly defined (with fewer than fifty cases having been described in the literature), as the symptoms are nonspecific and there is no straightforward method to confirm the diagnosis [4,5].

Unexplained pain in women is a common phenomenon, with many cases going unsolved. Historically, women are more likely to experience chronic pain conditions when compared to men and report more severe pain [6]. Despite this, women continue to struggle with having their pain recognized and properly worked up by healthcare providers, likely as a result of gender bias and the false attribution of pain to hormonal or psychological causes. For example, somatic symptom disorder, a psychological condition in which there is an extreme focus on physical symptoms (such as pain) that cause significant distress and problems functioning, is diagnosed 10 times more frequently in women than men [7]. Conditions such as these may be overdiagnosed in women due to the biases described above, with more serious and life-threatening disorders, such as DDS, being underdiagnosed. The purpose of this exploration is to shed light on DDS as an explanation for this female patient’s unresolved pain and to create greater awareness of this condition and its effect on dialysis patients. This case highlights a significant gap by demonstrating that DDS can present with severe pain in a patient lacking many of the classic risk factors, therefore underscoring the need for broader clinical awareness and consideration of this diagnosis.

## Case presentation

The case involves a 27-year-old Hispanic female with a past medical history of type 1 diabetes mellitus (T1DM), diabetic gastroparesis, hypertension, recurrent pyelonephritis, hyperlipidemia, depression, and end-stage renal disease (ESRD) secondary to poorly controlled T1DM on dialysis who presented to the emergency department with a three-day history of 10/10 severe left flank pain and right upper quadrant (RUQ) pain, abdominal distension, nausea, vomiting, bilateral lower extremity swelling, and headache. She stated she had been getting similar pain to this over the past four months, shortly after she began receiving dialysis for ESRD.

The patient’s family history included a mother with hyperthyroidism, a maternal grandfather with heart disease and myocardial infarctions, and a paternal great-grandmother with diabetes. The patient was born and raised in Cuba and immigrated to Miami 18 months prior to her admission. She was not employed and had neither a spouse nor children. She was sexually active with one male partner, using condoms for contraception, with no history of sexually transmitted infections. She denied the use of alcohol, tobacco, and illicit drugs. The patient's long-term medications provided at the time of admission are outlined in Table 1.

**Table 1:** Patient’s long-term medications provided at the time of admission PO: per os (by mouth, orally), SubQ: subcutaneous (under the skin), BID: twice daily (bis in die), MWF: Monday, Wednesday, Friday, QD: once daily (quaque die), QHS: every night at bedtime (quaque hora somni), QAM: every morning (quaque ante meridiem), TIDAC: three times daily before meals (ter in die ante cibum), TU, TH, SAT: Tuesday, Thursday, Saturday, TID: three times daily (ter in die)

Medication	Dose	Route	Frequency
Amoxicillin-clavulanate	875-125 mg	PO	BID
Bumetanide	1 mg	PO	BID
Calcitriol	0.25 mcg	PO	MWF
Famotidine	20 mg	PO	BID
Ferrous sulfate	325 mg	PO	QD
Flomax	0.4 mg	PO	QHS
Gabapentin	100 mg	PO	QD
Insulin glargine solostar pen	100 units/mL	SubQ	QAM
Insulin glargine	9 units	SubQ	QHS
Insulin lispro	4 units	SubQ	TIDAC
Labetalol	200 mg	PO	BID
Nifedipine	60 mg	PO	BID
Oxybutynin	5 mg	PO	BID
Retacrit	3000 m/L	SubQ	TU, TH, SAT
Senna	8.6 mg	PO	QHS
Sertraline	50 mg	PO	QD
Sevelamer carbonate	800 mg	PO	TID

The patient had missed a dialysis session and was presenting with signs of fluid overload (mild abdominal distension, lower extremity swelling), nausea, vomiting, left flank pain, hypertensive emergency, metabolic acidosis, and uremia. Nephrology was consulted, and emergent hemodialysis was completed. She was admitted to the Internal Medicine team for further work-up and treatment of left flank and RUQ pain, and to receive hemodialysis while inpatient. The patient's physical examination on admission is outlined in Table 2.

**Table 2:** Patient's physical exam on admission HEENT: head, eyes, ears, nose, and throat, RUQ: right upper quadrant, LCVA: left costovertebral angle

Physical exam
General: The patient appeared uncomfortable and in severe distress secondary to pain.
HEENT: Normocephalic and atraumatic. No scleral icterus. No oropharyngeal abnormalities.
Neck: No jugular venous distension or lymphadenopathy.
Cardiovascular: Regular rate and rhythm, no murmurs, rubs, or gallops. Peripheral pulses palpable. No evidence of peripheral cyanosis.
Respiratory: Lungs clear to auscultation bilaterally without wheezes, rales, or rhonchi. No increased work of breathing.
Abdomen: Mild abdominal distension present. Severe tenderness to palpation in the RUQ with moderate generalized abdominal tenderness. LCVA tenderness was noted. No rebound or guarding. Bowel sounds present.
Extremities: Bilateral lower extremity swelling. No clubbing or cyanosis.
Skin: No rash or lesions. No jaundice or pallor.
Neurological: Alert and oriented ×3, no focal neurological deficits. Cranial nerves II–XII grossly intact.

Of note, the patient was recently discharged four days prior for treatment of pyelonephritis (with concurrent grade 2 hydronephrosis of the left kidney). The patient presented on this current admission with a blood pressure of 194/97 and anion gap metabolic acidosis (pH 7.32, pCO2 36, HCO3 18, AG 17) in the emergency department. Venous blood gas and metabolic lab findings are presented in Table 3. Urinalysis results were as follows: 300 protein, 300 glucose, trace ketones, 0.03 blood, 75 leukocyte esterase, and 58 WBC (improved from prior admission).

**Table 3:** Patient's labs on admission pH: potential of hydrogen (measure of acidity/alkalinity), PCO2: partial pressure of carbon dioxide, pO2: partial pressure of oxygen, HCO3: bicarbonate, CO2: carbon dioxide, BUN: blood urea nitrogen

Venous blood gas		Normal range
pH	7.32 (low)	7.35-7.45
PCO2	36 (low)	35-45 mmHg
pO2	85	80-100 mmHg
HCO3	18 (low)	22-28 mEq/L
Metabolic panel		
Glucose	346 (high)	70-100 mg/dL
Sodium	131 (low)	135-145 mEq/L
Potassium	6.6* (hemolyzed)	3.7-5.2 mEq/L
Chloride	105	96-106 mEq/L
CO2	12 (low)	23-29 mEq/L
Anion gap	14	8-16 mEq/L
Osmolality	301 (high)	275-295 mOsm/kg
BUN	82 (high)	6-20 mg/dL
Creatinine	5.57 (high)	0.6-1.3 mg/dL
Calcium	9	8.5 and 10.2 mg/dL
Total protein	8.1	6.0-8.3 g/dL
Albumin	4.4	3.4 to 5.4 g/dL

Pyelonephritis was also on the differential, as she had recently been admitted for pyelonephritis and continued to have similar left flank pain with left costovertebral angle (LCVA) tenderness on physical exam. However, the patient was afebrile, did not have leukocytosis, was compliant with antibiotics after discharge, and urinalysis showed improvement in leukocyte esterase and WBCs. The US retroperitoneal showed no signs of hydronephrosis. It was therefore unlikely that inadequately treated pyelonephritis was the cause of her pain. At that time, the team continued to empirically treat for pyelonephritis as well as other abdominal infectious etiologies due to her severe pain.

**Figure 1:**
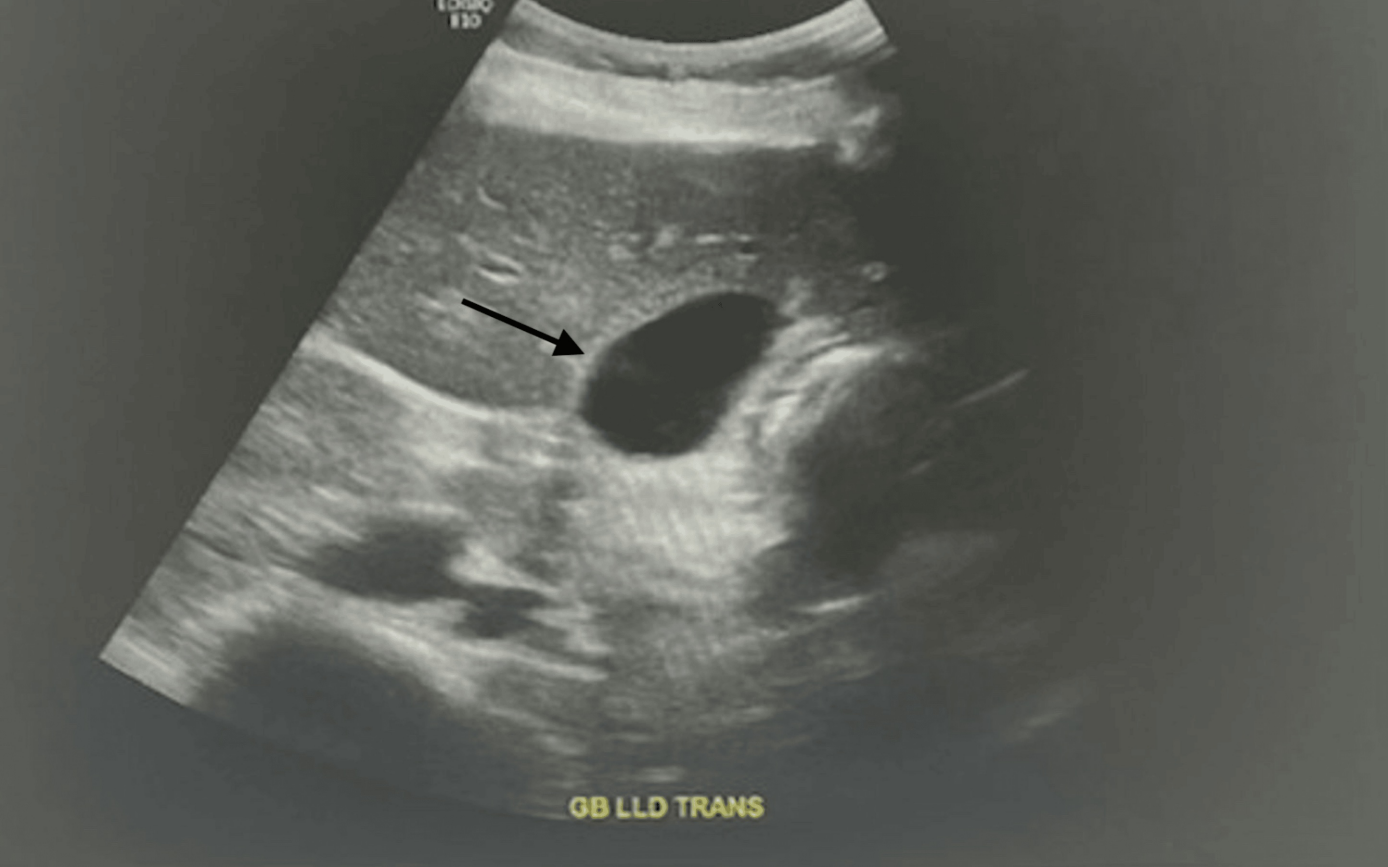
US abdominal Trace gallbladder sludge with borderline thickened gallbladder wall, no pericholecystic fluid, and negative sonographic Murphy sign; these findings are equivocal for acute cholecystitis. The follow-up HIDA scan was negative. US: ultrasound, HIDA: hepatobiliary iminodiacetic acid

The US retroperitoneal was negative for acute pyelonephritis, and the US abdominal (Figure 1) was equivocal for acute cholecystitis. The acute care surgery team was consulted to evaluate for cholecystitis. However, given the chronic nature of this pain, lack of fever, and equivocal imaging findings, there was lower concern for cholecystitis. Other differentials for RUQ pain included cholelithiasis or biliary colic, although this was less likely, as the pain was not postprandial and there were no corresponding imaging findings. The patient had chronic dyspepsia and constipation, which could have also worsened the underlying etiology.

After emergent dialysis, the patient’s vital signs stabilized, and signs of fluid overload improved. On the following day, however, she continued to report 10/10 generalized abdominal pain, requiring morphine every six hours without relief. She endorsed headache, nausea, upper chest pain, and palpitations. On physical exam, she appeared uncomfortable and in severe pain, with severe RUQ tenderness to palpation, moderate generalized abdominal tenderness, and LCVA tenderness.

**Figure 2:**
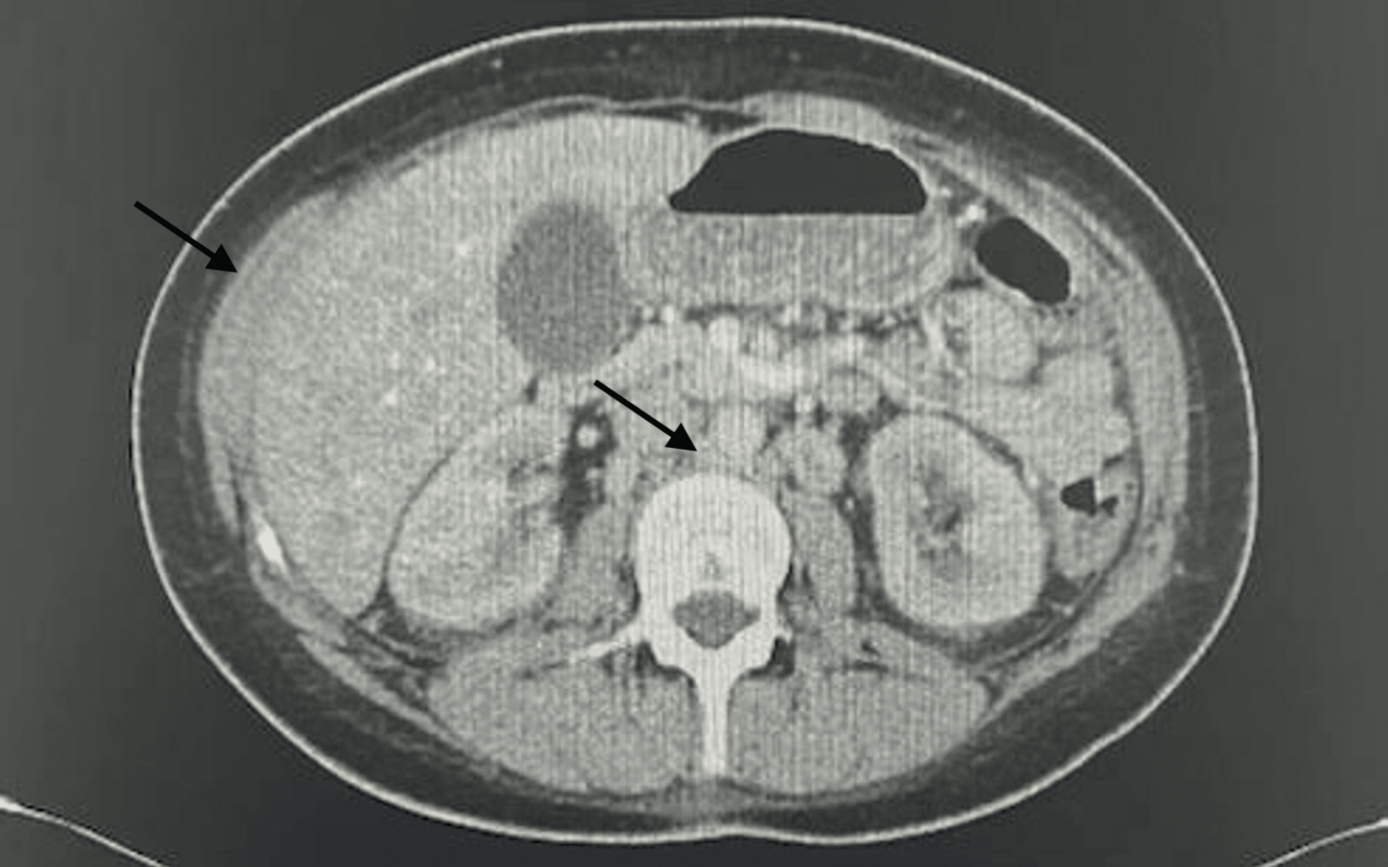
CT abdomen/pelvis Kidneys with normal enhancement, no hydronephrosis, similar left peri-aortic lymphadenopathy, new mild focal stranding in the right paracolic gutter, nonspecific.

Given that the patient’s abdominal pain began four months ago when she started dialysis and worsened after receiving dialysis, along with associated nausea, vomiting, and chest pain, her symptoms appeared consistent with DDS. A CT abdomen was ordered to rule out other causes, which showed similar left peri-aortic lymphadenopathy compared to the prior CT and new mild, nonspecific focal stranding in the right paracolic gutter (Figure 2), with no other acute abnormalities. Lipase and amylase were within normal limits, making pancreatitis unlikely. Urine and blood cultures showed no growth.

An EKG was ordered given new chest pain and palpitations, which showed prolonged QTc at 518 ms. Ondansetron was discontinued due to prolonged QTc, and scopolamine was started for nausea instead. The patient continued to have prolonged QTc, despite switching from ondansetron to scopolamine and ruling out other causes (electrolyte disturbances, hypothyroidism, and congenital). All other medications were reviewed and not associated with QTc prolongation. However, dialysis can be associated with prolonged QTc and may be an explanation for this finding [8]. The repeat EKG continued to show a prolonged QTc of 508 ms, notably after dialysis.

**Figure 3:**
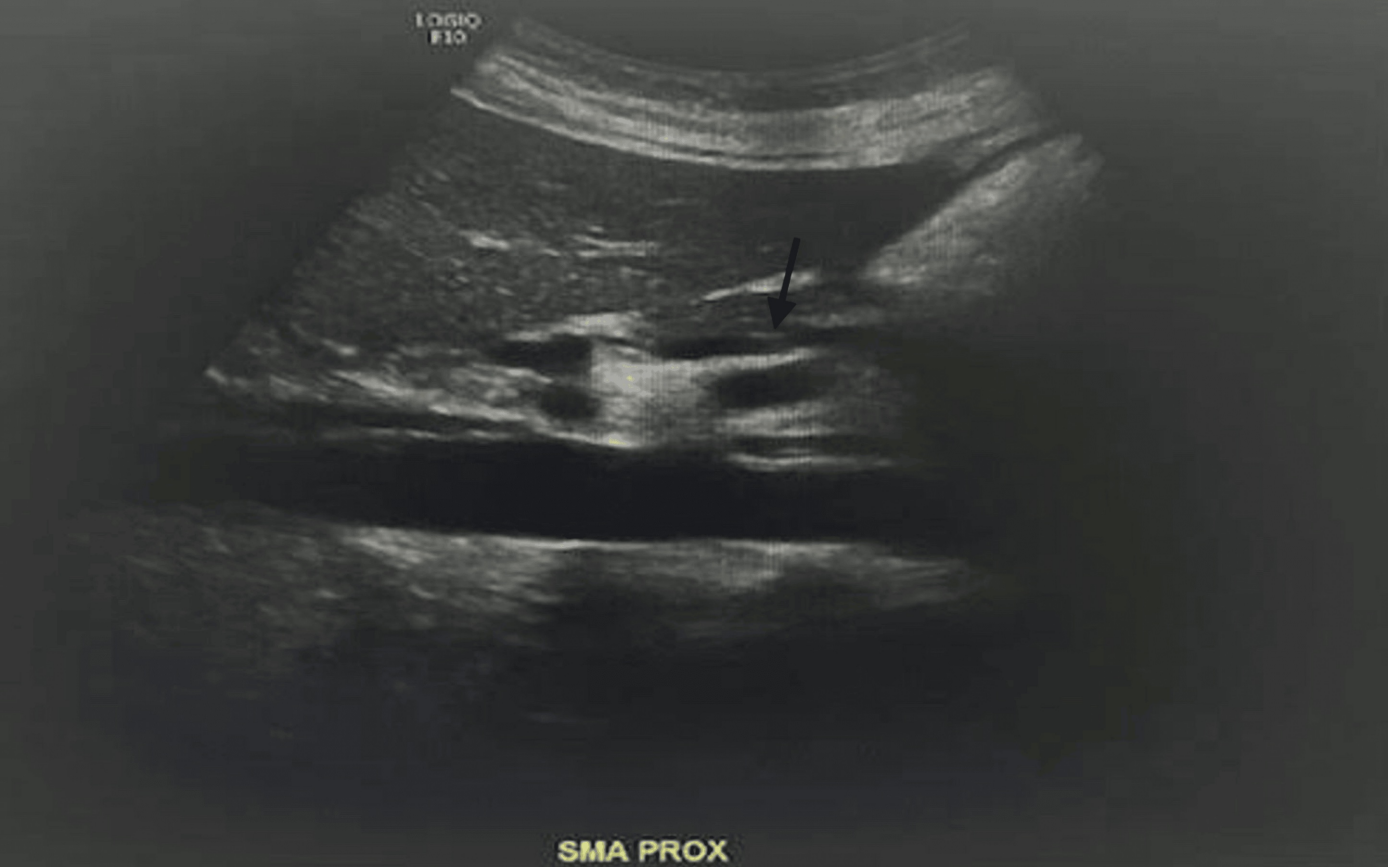
US duplex mesenteric arteries No evidence of significant stenosis in the mesenteric arteries. US: ultrasound

The next day, the patient stated her pain was 4/10 in severity and was improving with hydromorphone 2 mg every four hours. She continued to be nauseous and vomited twice. On the third day of admission, the patient received an additional dialysis session in the morning. In the afternoon, the patient was in severe 10/10 pain again, appearing extremely uncomfortable. She endorsed pain in the substernal region, diffuse abdomen, and back. Due to the patient's pain being out of proportion to the exam and the high risk of mesenteric ischemia given her history of diabetes, gastroparesis, and dialysis sessions, a lactate level and US duplex of the mesenteric arteries were ordered. The lactate was within normal limits, and the US duplex showed patent mesenteric arteries (Figure 3).

**Figure 4:**
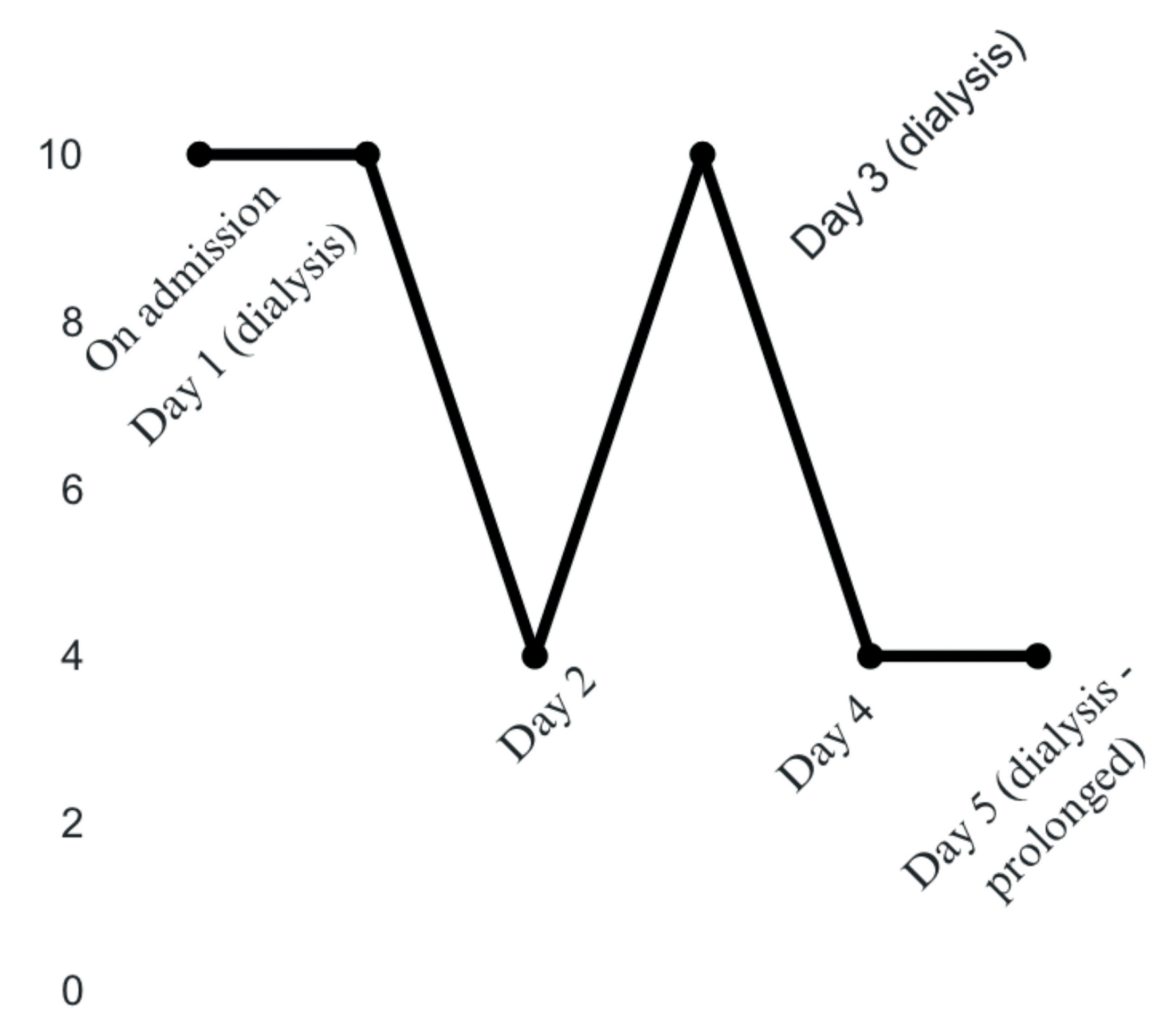
Patient’s pain level throughout hospital admission

Due to suspicion of DDS, the patient was recommended to elongate dialysis sessions and minimize interruptions to the dialysis schedule, which provided minimal improvement in pain. The following day, after the patient’s pain had decreased to 4/10, she was discharged from the hospital. The patient followed up in the clinic after discharge and noted improvement in her pain and symptoms after modifications to her dialysis were made. The patient's pain level throughout hospital admission is illustrated in Figure 4.

Differential diagnoses and investigations for abdominal pain in dialysis patients are described in Table 4.

**Table 4:** Differential diagnoses and investigations for abdominal pain in dialysis patient US: ultrasound, CT: computed tomography, DDS: dialysis disequilibrium syndrome

Differential diagnosis	Rationale for consideration	Study/findings	Outcome
Cholecystitis	RUQ pain and abdominal tenderness	US abdomen: equivocal for cholecystitis, HIDA scan: negative	Lower concern, ruled out due to lack of fever and imaging findings
Cholelithiasis/biliary colic	RUQ pain	US abdomen: no evidence of gallstones	Ruled out due to non-postprandial pain and lack of imaging findings
Pancreatitis	Abdominal pain, nausea, vomiting	CT abdomen: negative, amylase/lipase: within normal limits	Ruled out due to normal imaging and enzyme levels
Pyelonephritis	Left flank pain, recent pyelonephritis diagnosis	Urinalysis: decreased leukocyte esterase/WBC from previous admission, US retroperitoneal: no hydronephrosis	Ruled out due to lack of fever, improvement in urine studies, and negative imaging findings
Fluid overload/uremia	Missed dialysis session, signs of fluid overload	Clinical observation: improved after hemodialysis	Temporarily treated, but ruled out as the primary cause of persistent pain
Mesenteric ischemia	Severe abdominal pain, diabetes, gastroparesis, dialysis	US mesenteric arteries: patent mesenteric arteries, lactate level: normal	Ruled out due to normal lactate levels and imaging
DDS	Persistent abdominal pain correlated with dialysis sessions	Clinical improvement: after prolonged hemodialysis	Most likely diagnosis

## Discussion

This patient struggled with a non-specific array of symptoms that originally did not point toward any one diagnosis. Throughout her hospital stay, disorders that could have explained her pain, such as cholecystitis, mesenteric ischemia, and ongoing pyelonephritis, were ruled out. Upon questioning the patient, it appeared that her severe pain, nausea, vomiting, and headaches would typically begin shortly after her dialysis sessions. Her symptoms would become even more severe when she missed dialysis sessions and subsequently completed her sessions a few days late, consistent with the diagnosis of DDS. DDS is thought to occur due to rapid osmotic shifts during hemodialysis, in which blood urea levels fall faster than urea can equilibrate across the blood-brain barrier. This creates an osmotic gradient that drives water into brain cells, leading to cerebral edema, which explains the constellation of neurological (headache, nausea, altered mentation) and systemic symptoms observed [2]. Electrolyte shifts, particularly in sodium and bicarbonate following dialysis, may further exacerbate these osmotic gradients and contribute to symptom severity [2], highlighting the multifactorial nature of DDS in this patient. The systemic symptoms, such as chest pain, palpitations, and abdominal pain, are thought to arise indirectly. Increased intracranial pressure can trigger activation of the autonomic nervous system, leading to dysregulated blood pressure, heart rate, and perceived chest pain or palpitations [9]. Additionally, when the brain is under stress due to edema, pain perception may be amplified through central sensitization [10].

This patient’s case was unique in that she lacked many of the risk factors typically associated with this syndrome. The patient’s BUN was <175 mg/dL, she was not on the extremes of age, and she did not have a pre-existing neurological condition. The patient believed her pain began a few months ago, coinciding with when she first began dialysis treatment. Initial dialysis treatment has been noted as one of the significant risk factors for this disorder. However, since DDS lacks definitive diagnostic criteria and this patient did not demonstrate all of the classic risk factors, the diagnosis was ultimately made by exclusion and clinical judgment, which should be acknowledged as a limitation when interpreting this case. This underscores the importance of maintaining DDS on the differential diagnosis in dialysis patients with unexplained symptoms, even when classic risk factors are absent. Due to the non-specific presentation and difficulty in confirming this syndrome, it is easily missed as a diagnosis, and the number of cases is thought to be severely under-reported [4], making it essential for physicians to be aware of this as a potential cause of pain in dialysis patients. This is also necessary, as the consequences of DDS may be severe in cases in which cerebral edema occurs, resulting in seizures, coma, and death [4].

Additionally, this case is meant to encourage physicians to dig deeper into the unexplained pain of patients, with a focus on women, whose pain has had a history of being ignored [5]. Physicians may be quick to attribute somatic symptoms such as headache, nausea, vomiting, and pain to mental stressors and/or psychiatric disorders [6]. The patient discussed in this case has a history of depression, a known risk factor for chronic pain. Although much of the work-up was negative for this patient, it was evident that she was severely uncomfortable and that her pain was not purely psychosomatic. Despite many previous admissions, there was no consensus on her diagnosis; the electronic medical record showed many repeated images and labs with no answers, simply ruling out the most common differentials. Patients such as these deserve for their providers to consider rarer causes of pain. In patients with ESRD, structured pain assessment frameworks can be helpful, such as the PEG scale (a three-item measure of pain intensity and interference with life) and KDQOL-36 (a kidney disease-specific quality of life survey) [11,12]. These tools enable providers to capture both the physical and psychosocial dimensions of pain, ensuring it is properly evaluated rather than dismissed.

Once DDS is suspected, several measures can be taken to avoid symptoms during and after dialysis sessions. The administration of hypertonic saline has been shown to reduce symptoms, such as muscle cramps and headaches [13]. Hemofiltration may also be used in place of conventional dialysis to avoid the rapid change in osmolalities of the body’s fluid compartments [14]. For patients continuing on standard hemodialysis, it is recommended to prolong sessions and limit the reduction in urea concentration to 40% over two hours, as the rapid fall in urea is thought to underlie many DDS symptoms [15]. To achieve this, the patient’s volume of distribution of urea can be estimated from body weight, allowing urea kinetic modeling to guide blood flow rate and dialysis duration [16]. Case series and reviews consistently recommend short, frequent hemodialysis sessions with lower blood and dialysate flow rates, limiting early urea reduction to around 30-40% to avoid rapid osmotic shifts [2,15]. In addition, sodium modeling or higher dialysate sodium concentrations have been described in case reports as effective in reducing symptoms such as nausea, headache, and cramping during subsequent dialysis sessions [17]. Other reports have noted improvement with the administration of mannitol or hypertonic saline, which act as osmotic agents to counter cerebral edema [18]. More recently, the use of sustained low-efficiency dialysis or continuous renal replacement therapy has been suggested for high-risk patients, as these modalities minimize osmotic shifts and have not been associated with DDS in published cohorts [19]. While these approaches are consistently recommended in reviews and expert guidance, most data remain observational, and randomized trials are still needed to establish standardized protocols and improve patient outcomes.

## Conclusions

This case report highlights the importance of considering DDS as a potential diagnosis in patients experiencing unexplained pain following hemodialysis, especially in the early months of treatment. This 27-year-old female patient presented with severe abdominal pain, headache, nausea, and vomiting that worsened after dialysis sessions, despite lacking many typical risk factors for DDS. Her symptoms were consistent with the clinical picture of DDS, which is often underreported and challenging to diagnose due to its nonspecific presentation. By raising awareness of DDS and its varied presentations, this case aims to improve the recognition and management of this syndrome in dialysis patients. Further research is needed to better understand the pathophysiology of DDS and develop more effective prevention and treatment strategies. Ultimately, increased awareness and proper management of DDS can lead to improved quality of life for dialysis patients and potentially prevent the severe complications associated with this syndrome.
